# Tetanus, Diphtheria, Pertussis Vaccination Coverage Before, During, and After Pregnancy — 16 States and New York City, 2011

**Published:** 2015-05-22

**Authors:** Indu B. Ahluwalia, Helen Ding, Denise D’Angelo, Kristen H. Shealy, James A. Singleton, Jennifer Liang, Kenneth D. Rosenberg

**Affiliations:** 1 Division of Reproductive Health, National Center for Chronic Disease Prevention and Health Promotion, CDC; 2 DB Consulting Group, Inc; 3 Immunization Services Division, National Center for Immunization and Respiratory Diseases, CDC; 4 Division of Bacterial Diseases, National Center for Immunization and Respiratory Diseases, CDC; 5 PRAMS Oregon, Maternal & Child Health Epidemiologist, Oregon Public Health Division

In June 2011, the Advisory Committee on Immunizations Practices (ACIP) recommended 1 dose of a tetanus, diphtheria, and acellular pertussis (Tdap) vaccine during pregnancy for women who had not received Tdap previously (1). Before 2011, Tdap was recommended for unvaccinated women either before pregnancy or postpartum (2). In October 2012, ACIP expanded the 2011 recommendation, advising pregnant women to be vaccinated with Tdap during each pregnancy to provide maternal antibodies for each infant (3). The optimal time for vaccination is at 27–36 weeks’ gestation as recommended by ACIP. In response to ACIP’s Tdap recommendation for pregnant women in 2011, CDC added a supplemental question to the Pregnancy Risk Assessment Monitoring System (PRAMS) survey to determine women’s Tdap vaccination status before, during, or after their most recent delivery. This report describes overall and state-specific Tdap vaccination coverage around the time of pregnancy using data from 6,852 sampled women who delivered a live-born infant during September–December 2011 in one of 16 states or New York City (NYC). Among the 17 jurisdictions, the median percentage of women with live births who reported any Tdap vaccination was 55.7%, ranging from 38.2% in NYC to 76.6% in Nebraska. The median percentage who received Tdap before pregnancy was 13.9% (range = 7.7%–20.1%), during pregnancy was 9.8% (range = 3.8%–14.2%), and after delivery was 30.9% (range = 13.6%–46.5%). The PRAMS data indicate a wide variation in Tdap vaccination coverage among demographic groups, with generally higher postpartum coverage for non-Hispanic white women, those who started prenatal care in the first trimester, and those who had private health insurance coverage. This information can be used for promoting evidence-based strategies to communicate the importance of ACIP guidelines related to Tdap vaccination coverage to women and their prenatal care providers.*

CDC analyzed data from PRAMS, an ongoing, population-based survey that collects data on maternal behaviors around the time of pregnancy from women who delivered a live-born infant. Approximately 2 months after delivery, the PRAMS program in each state takes stratified random samples of 100–300 women from each site’s birth registry. The selected mothers were mailed up to three questionnaires, and those who did not respond by mail were contacted by telephone. For mothers who delivered a live-born infant during September–December 2011 and were surveyed during December 2011–May 2012, a questionnaire supplement with a question about Tdap vaccination status was included. PRAMS data collected by 16 states and New York City were analyzed. All states included in the analysis met the PRAMS response threshold of 65% (median = 69.5%; range = 65.0%–81.0%); states with less than 65% response were not included in the analysis. Weighted PRAMS data for 2011 were aggregated, and Tdap vaccination coverage was estimated for each of the 16 states and NYC. In addition, for the aggregate of states, both overall Tdap vaccination coverage and overall coverage before, during, and after pregnancy were reported. Tdap vaccination coverage was examined by selected characteristics for aggregated data. Data from respondents who reported not knowing whether or not they had received Tdap vaccination were excluded from estimates of Tdap vaccination coverage. All estimates were weighted to account for the complex survey design and nonresponse.

As an additional analysis, information collected by Oregon PRAMS during 2009–2011 on whether women’s providers offered them a Tdap vaccination postpartum was examined by CDC. The Oregon PRAMS survey did not ask about Tdap vaccination status during 2011.

Overall, of the 6,852 women who delivered a live-born infant and responded to the Tdap question in PRAMS, 20.8% (1,353) did not know their vaccination status. Among the 5,499 with known vaccination coverage status overall, 53.4% reported being vaccinated with Tdap, including 13.9% before pregnancy, 9.9% during pregnancy, and 30.5% after delivery. There was wide variation in Tdap vaccination coverage by jurisdiction among respondents with a median of 55.7% (range = 38.2%–76.6%) (Figure) (Table 1). PRAMS data also indicated higher postpartum Tdap vaccination prevalence among non-Hispanic white women, those with private insurance, and those who initiated prenatal care in the first trimester (Table 2). In Oregon, the percentage of women who reported that their provider offered Tdap vaccination postpartum increased from 30.3% (95% confidence interval [CI] = 26.9%–33.9%) in 2009 to 47.1% (CI = 43.7%–50.5%) in 2010 to 55.8% (CI = 52.3%–59.2%) in 2011 (p<0.05).

## Discussion

Before the June 2011 change in the Tdap recommendation for pregnant women, postpartum vaccination was recommended by ACIP and the American College of Obstetricians and Gynecologists. Results from this analysis might reflect the early transition from a policy of vaccinating women postpartum to a policy of vaccinating them during pregnancy (1). Among PRAMS participants, over half reported having been vaccinated at some time before, during, or after pregnancy and of these, most reported being vaccinated after delivery. Overall coverage varied both among states and by demographic groups as seen previously among U.S. adults (4). Two studies using different data sets, one using Vaccine Safety Datalink information from large, private, medical care organizations and another using Medicaid claims data, show results similar to PRAMS in that those reporting early entry into prenatal care and non-Hispanic whites were more likely to report being vaccinated (5,6). The differences in timing of coverage reported in these studies and PRAMS might be the result of methodologic differences in data sources and ascertainment.

PRAMS data on Tdap, similar to influenza vaccination coverage, indicate that Tdap vaccination coverage was lower for non-Hispanic black women, those with Medicaid health care coverage for prenatal care, and those starting prenatal care after the first trimester of pregnancy. To improve coverage, coordinated, cross-sector efforts are needed, similar to those that occurred during the 2009 influenza A (H1N1) pandemic. These included vaccination promotion to providers and patients, removal of reimbursement barriers, coordination, and communication during the pandemic (7–9). Previous research on seasonal influenza vaccination shows that health care provider recommendations are strongly associated with a higher coverage among pregnant women; similar vaccination recommendations by providers might be needed to reach greater Tdap coverage (10). In addition, variation in coverage might have occurred as a result of state-specific programs and policies on adult immunizations.

The findings in this report are subject to at least five limitations. First, PRAMS data are self-reported several months after delivery and are subject to recall bias. Second, the response rates varied among the states from 65% to 81% and might be subject to nonresponse bias even after weighting adjustment. Third, 20.8% of respondents did not know their Tdap vaccination status, and overall aggregate estimates of Tdap vaccination could have ranged from 43.1% to 63.9% depending on whether none or all of those reporting unknown Tdap vaccination status were vaccinated. Fourth, reported Tdap vaccinations after pregnancy could have occurred any time during the period immediately after delivery until the date the survey was completed, and do not necessarily indicate immediate postpartum vaccination given at the birthing facility. The optimal time for vaccination is at 27–36 weeks’ gestation as recommended by ACIP. Finally, data were available for a 4-month period and thus do not represent the entire year of data collection among women with live-born infants; the results are not generalizable to all women delivering a live-born infant in the states included in this analysis.

What is already known on this topic?

Infants have substantially higher rates of pertussis and the largest burden of pertussis-related deaths. Maternal vaccination with tetanus, diphtheria, and acellular pertussis (Tdap) vaccine protects infants from pertussis. The Advisory Committee on Immunization Practices (ACIP) recommended in 2012 that pregnant women be vaccinated with Tdap during each pregnancy regardless of previous immunization status. Women who are not vaccinated during pregnancy should be vaccinated with Tdap during the postpartum period.

What is added by this report?

Among 16 states and New York City participating in the Pregnancy Risk Assessment Monitoring System supplemental data collection, the median proportion of women with recent live-births during September–December 2011 who reported receiving Tdap vaccination before pregnancy was 13.9%, during pregnancy was 9.8%, and after delivery was 30.9%. These results can provide a baseline for evaluating implementation of the current recommendations for Tdap vaccination for pregnant women.

What are the implications for public health practice?

Efforts to promote and educate pregnant women and their providers on the importance of Tdap vaccination during pregnancy are needed to increase coverage of Tdap among pregnant women to protect mothers and their infants from pertussis.

With almost one fifth of women not knowing their Tdap vaccination status, there is a widespread need for providers to ensure they are communicating information about recommended vaccinations and to educate all women about the importance of keeping their vaccination status up-to-date and documented, especially reproductive-age women (5). Health care providers can assist pregnant women by providing specific information about where to obtain Tdap vaccination, or offering to provide the vaccination, and also to write a prescription in case it is needed; additional tools for providers are available.† Knowledge of Tdap vaccination among women and health care providers might be lagging because the changes to the Tdap recommendation were relatively recent (1,3). Promoting communication strategies that increase awareness of Tdap recommendations to providers, pregnant women, adults, and anyone who might come into contact with infants aged <12 months is important.

Estimates from Oregon indicate that the proportion of providers who offered Tdap vaccination postpartum increased from 2009 to 2011, likely reflecting adoption of 2005 recommendations for pregnant women (2). Shortly after being recommended during pregnancy in June 2011, Tdap vaccination coverage during pregnancy was low; however, those results might reflect only the first few months of full implementation of the recommendation, which was published by CDC in October 2011. In contrast, this report assessed coverage among women delivering a live-born infant during September–December 2011. Thus, results in this report might provide a baseline for evaluating implementation of the current Tdap recommendations for pregnant women.

## Figures and Tables

**FIGURE:**
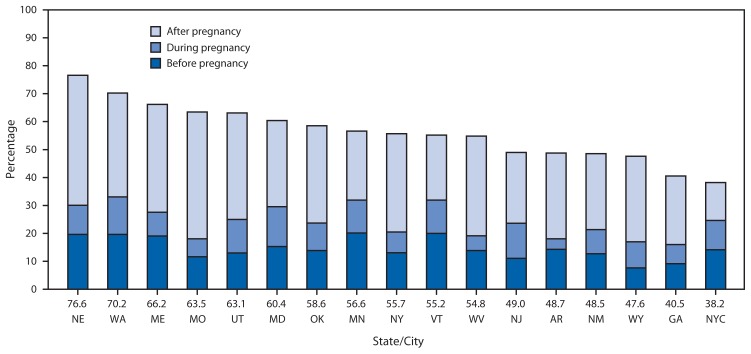
Percentage of women reporting receiving Tdap before, during, and after pregnancy among those delivering a live-born infant during September–December 2011, by state/city — Pregnancy Risk Assessment Monitoring System, 16 states and New York City, 2011 **Abbreviation:** Tdap = tetanus, diphtheria, and acellular pertussis vaccine. **State/city abbreviations:** AR = Arkansas; GA = Georgia; MD = Maryland; ME = Maine; MN = Minnesota; MO = Missouri; NE = Nebraska; NJ = New Jersey; NM = New Mexico; NY = New York; NYC = New York City; OK = Oklahoma; UT = Utah; VT = Vermont; WA = Washington; WV = West Virginia; WY = Wyoming.

**TABLE 1:** Percentage of women reporting Tdap vaccination before, during, and after pregnancy among those who had a live birth during September–December 2011, by state/city — Pregnancy Risk Assessment Monitoring System, 16 states and New York City, 2011

State/City	No. in sample*	Vaccinated with Tdap							

		Overall		Before pregnancy		During pregnancy		After pregnancy	
			
		Weighted %	(95% CI)	Weighted %	(95% CI)	Weighted %	(95% CI)	Weighted %	(95% CI)
**Overall**	**5,499**	**53.3**	**(51.3–55.4)**	**13.9**	**(12.6–15.3)**	**9.7**	**(8.5–10.9)**	**29.8**	**(28.0–31.7)**
Arkansas	241	48.7	(38.4–59.2)	14.3	(8.2–23.8)	3.8	(1.5–9.4)	30.7	(21.9–41.0)
Georgia	329	40.5	(31.9–49.8)	9.2	(5.1–15.8)	6.8	(3.5–12.9)	24.6	(17.5–33.3)
Maryland	378	60.4	(52.6–67.7)	15.3	(10.4–21.9)	14.2	(9.4–21.0)	30.9	(24.2–38.6)
Maine	278	66.2	(59.3–72.4)	19.1	(14.4–24.9)	8.5	(5.1–13.9)	38.6	(32.2–45.4)
Minnesota	363	56.6	(50.9–62.2)	20.1	(16.1–24.9)	11.8	(8.6–16.1)	24.7	(20.2–29.9)
Missouri	299	63.5	(57.1–69.4)	11.6	(8.1–16.4)	6.4	(3.9–10.5)	45.4	(39.2–51.8)
Nebraska	447	76.6	(71.7–80.8)	19.7	(15.6–24.5)	10.4	(7.6–14.0)	46.5	(41.1–52.1)
New Jersey	383	49.0	(43.5–54.5)	11.1	(8.0–15.2)	12.6	(9.4–16.7)	25.4	(20.9–30.4)
New Mexico	379	48.5	(43.6–53.5)	12.7	(9.8–16.5)	8.7	(6.2–12.0)	27.2	(23.0–31.8)
New York	260	55.7	(48.0–63.2)	13.1	(8.7–19.1)	7.4	(4.2–12.7)	35.2	(28.3–42.8)
Oklahoma	390	58.6	(49.9–66.7)	13.9	(8.8–21.3)	9.8	(5.5–17.0)	34.8	(27.3–43.2)
Utah	334	63.1	(57.1–68.7)	13	(9.5–17.5)	12	(8.5–16.6)	38.1	(32.4–44.2)
Vermont	253	55.2	(48.9–61.3)	20	(15.5–25.4)	12	(8.4–16.7)	23.3	(18.5–28.9)
Washington	292	70.2	(63.5–76.2)	19.6	(14.7–25.7)	13.4	(9.5–18.7)	37.2	(30.6–44.2)
West Virginia	420	54.8	(49.0–60.5)	13.8	(10.2–18.4)	5.3	(3.3–8.5)	35.7	(30.2–41.5)
Wyoming	145	47.6	(38.4–57.0)	7.7	(4.0–14.1)	9.3	(5.1–16.5)	30.7	(22.8–39.9)
NYC	308	38.2	(31.6–45.4)	14.2	(9.9–19.9)	10.4	(6.8–15.7)	13.6	(9.4–19.3)
**Median**	**329**	**55.7**		**13.9**		**9.8**		**30.9**	
**Maximum**	**447**	**76.6**		**20.1**		**14.2**		**46.5**	
**Minimum**	**145**	**38.2**		**7.7**		**3.8**		**13.6**	

**Abbreviations:** CI = confidence interval; Tdap = tetanus, diphtheria, and acellular pertussis.

* Excluded those who reported “don’t know” or “missing” for their vaccination status (n = 1,353).

**TABLE 2:** Percentage of women reporting Tdap vaccination before, during, and after pregnancy among those who had a live birth during September–December 2011, by selected characteristics — Pregnancy Risk Assessment Monitoring System, 16 states and New York City, 2011

Characteristic	No. in sample*	Vaccinated with Tdap					

		Before pregnancy		During pregnancy		After pregnancy	
		
		Weighted %	(95% CI)	Weighted %	(95% CI)	Weighted %	(95% CI)
**Age group (yrs)**							
<20†	452	13.5	(9.0–19.7)	16.2	(11.1–22.8)	26.7	(20.4–34.1)
20–24	1,221	11.3	(8.7–14.7)	11.9	(9.2–15.3)	28.0	(24.0–32.5)
25–29	1,512	14.4	(12.0–17.3)	9.2	(7.1–11.8)	33.3	(30.0–37.2)
30–34	1,363	15.9	(13.2–19.0)	8.3§	(6.4–10.7)	33.2	(29.5–37.2)
≥35	949	13.6	(10.5–17.5)	9.0§	(6.4–12.3)	26.2	(22.3–30.6)
**Race/Ethnicity**							
Hispanic	767	12.6	(9.6–16.5)	17.1§	(13.6–21.2)	21.2§	(18.3–26.6)
White, non-Hispanic†	3,281	15.0	(13.2–17.0)	7.5	(6.2–9.1)	34.9	(32.4–37.6)
Black, non-Hispanic	748	10.7	(7.4–15.2)	12.8	(9.0–18.0)	21.7§	(16.7–27.6)
Other	690	14.2	(10.8–18.3)	9.8	(7.0–13.4)	28.0	(23.1–33.6)
**Marital status**							
Married†	3,338	15.8	(14.0–17.8)	7.4	(6.1–8.8)	32.6	(30.2–35.1)
Other	2,151	10.8§	(8.8–13.1)	14.2§	(11.9–16.9)	27.1§	(24.1–30.4)
**Education**							
<High school†	782	12.1	(8.8–16.4)	14.7	(11.1–19.3)	21.2	(16.9–26.3)
High school	1,331	8.5	(6.4–11.1)	13.2	(10.4–16.6)	30.8§	(26.6–35.5)
>High school	3,338	16.4	(14.6–18.4)	7.6§	(6.4–9.0)	32.9§	(30.5–35.3)
**Parity**							
One†	2,365	14.0	(12.0–16.2)	11.0	(9.1–13.1)	33.4	(30.5–36.6)
Two or more	3,092	14.0	(12.2–16.1)	9.0	(7.6–10.8)	28.5	(26.0–31.0)
**WIC-during pregnancy**							
No†	2,890	16.4	(14.5–18.5)	7.5	(6.2–9.1)	33.5	(31.0–36.2)
Yes	2,568	11.2§	(9.3–13.4)	13.0§	(10.9–15.3)	26.8§	(24.0–29.9)
**Health Insurance status at delivery**							
Private†	2,352	17.7	(15.5–20.2)	7.2	(5.8–8.9)	35.2	(32.4–38.1)
Medicaid	2,360	10.7§	(8.8–12.9)	12.5§	(10.5–14.9)	26.4§	(23.5–29.6)
Both	277	11.8	(6.1–21.7)	10.5	(5.9–18.0)	33.4	(23.6–44.7)
Other	375	12.3	(8.3–17.8)	13.2	(8.6–19.6)	28.0	(21.5–35.6)
**Prenatal care**							
First trimester†	4,509	14.5	(12.9–16.2)	9.8	(8.5–11.2)	32.2	(30.0–34.4)
Second trimester or later	825	11.6	(8.6–15.3)	10.9	(7.9–14.9)	23.5§	(19.1–28.5)

**Abbreviations:** CI = confidence interval; Tdap = tetanus, diphtheria, and acellular pertussis; WIC = Women, Infants, and Children Special Supplemental Nutrition Program.

* Excluded those who reported “don’t know” or “missing” for their vaccination status.

† Referent group.

§ p<0.05 compared with referent group.

## Footnotes

* Additional information available at http://www.thecommunityguide.org/vaccines/index.html.

† Additional information available at http://www.cdc.gov/pertussis/pregnant/HCP.
